# Human Part Segmentation in Depth Images with Annotated Part Positions

**DOI:** 10.3390/s18061900

**Published:** 2018-06-11

**Authors:** Andrew Hynes, Stephen Czarnuch

**Affiliations:** Department of Electrical and Computer Engineering, Memorial University, St. John’s, NL A1B 3X5, Canada; sczarnuch@mun.ca

**Keywords:** human parts, interactive image segmentation, occlusion, grid graph

## Abstract

We present a method of segmenting human parts in depth images, when provided the image positions of the body parts. The goal is to facilitate per-pixel labelling of large datasets of human images, which are used for training and testing algorithms for pose estimation and automatic segmentation. A common technique in image segmentation is to represent an image as a two-dimensional grid graph, with one node for each pixel and edges between neighbouring pixels. We introduce a graph with distinct layers of nodes to model occlusion of the body by the arms. Once the graph is constructed, the annotated part positions are used as seeds for a standard interactive segmentation algorithm. Our method is evaluated on two public datasets containing depth images of humans from a frontal view. It produces a mean per-class accuracy of 93.55% on the first dataset, compared to 87.91% (random forest and graph cuts) and 90.31% (random forest and Markov random field). It also achieves a per-class accuracy of 90.60% on the second dataset. Future work can experiment with various methods for creating the graph layers to accurately model occlusion.

## 1. Introduction

Segmenting the parts or limbs of a human body has applications for pose estimation [1], robotic assistance [2] and gaming [3]. Recent research on human limb segmentation has used machine learning techniques to perform per-pixel classification of RGB (colour) and/or depth images, without any pre-annotation. However, these methods often require manually segmented images for training and testing. Systems for pose estimation and recognition also make use of manually segmented images. For large datasets, the manual annotation of human limbs becomes laborious. This paper introduces a method for assisting with the semi-supervised segmentation of humans in depth images. A user specifies one (x,y) position for each body part in a depth image, and our system classifies each pixel on the body with an appropriate label.

In this problem, we are provided a depth image, a foreground segmentation of the human figure and a set of image positions for the body parts. The objective is to assign each pixel in the foreground to a body part. Our approach is to use the part positions in conjunction with an interactive image segmentation algorithm.

Interactive image segmentation involves an *n*-dimensional image, which is classified into *k* distinct regions. A user specifies seed pixels (or voxels) in regions of interest that are given a label from one to *k*. The regions are segmented using the values of the pixels. In this application, *k* is the number of body parts, and the pixels have only depth values.

While interactive segmentation algorithms can segment an image into logical regions, it can be difficult to apply them to human part segmentation, due to the presence of self-occlusion: when some regions of the body are occluded by others. In response, we use a graph representation that is designed to handle occlusion. The assumption is made that the arms are the only regions that can potentially occlude other parts. We create a grid graph with one to three layers: representing the base body, the right arm and the left arm. In this formulation, a single pixel in the image can be represented by more than one node in the graph. A standard interactive segmentation algorithm can then be applied to this layered graph, resulting in a more accurate segmentation of human limbs.

The paper is organized as follows. Section 2 discusses recent work towards human limb segmentation and provides an overview of some standard interactive image segmentation algorithms. Section 3 describes the process of creating a layered graph representation of a human depth image and determining seed nodes and labels that are used for interactive segmentation. Section 4 presents experimental results on two public datasets of human depth images [4,5]. The conclusions are presented in Section 5.

## 2. Related Works

### 2.1. Human Limb Segmentation

Relevant literature commonly refers to segmenting regions of the body as human limb segmentation. We refer to our system as human part segmentation, to make the distinction between limbs (e.g., arm) and parts (e.g., shoulder, elbow and hand).

Segmenting human limbs has been applied to single RGB images [6,7], RGB video sequences [8] and images combining colour and depth, referred to as RGB-D [2,9]. It has also been performed on pure depth images and videos [4,10].

Human limb segmentation from RGB-D data has seen recent application to mobility assistance robots [2]. Using RGB-D images provided by a Kinect camera, the limbs are segmented with a deep learning approach. The researchers also developed a method to abbreviate the manual per-pixel annotation of limbs. By computing histogram of oriented gradient (HOG) features on each image, they were able to cluster images using pairwise distances in HOG space. One image would be randomly selected from the cluster and manually segmented using an image editing tool. The segmentation would then be propagated to the other images in the cluster.

The authors in [4] introduced a general method for object segmentation in depth images and applied this to human limb segmentation. Building on the pose estimation algorithm developed for the Kinect [11], per-pixel classification was performed with a learned random forest classifier. The classification was further optimized using graph cuts and tested on a new dataset of RGB and depth images with per-pixel segmented limbs. This approach built off their earlier work [12], which performed limb segmentation with an interacting user, focusing on correcting user inputs using graph cuts.

The work of [10] involved segmenting parts of the hand in a depth image. Similar to [4], a random forest was used for the initial per-pixel classification. Instead of graph cuts, the pixel classification was optimized by partitioning the hand into superpixels and smoothing with a Markov random field. The method was shown to generalize to human part segmentation by testing on the dataset from [4].

The algorithms of [2,4,10] all required segmented datasets for training. The intention of our work is to assist with creating these datasets using interactive segmentation. While [12] used an interactive segmentation algorithm for human limbs, they used a standard grid graph structure to represent the image. Our main contribution is the proposal of a layered graph designed to handle occlusion, which can be segmented with an interactive algorithm.

### 2.2. Interactive Image Segmentation

The following algorithms were developed for general interactive image segmentation. They all share the properties of operating on a grid structure, such as a graph or lattice. The segmentation results depend on user-defined seeds and labels and the difference in value between pixels in the same local neighbourhood.

#### 2.2.1. Graph Cuts

The original graph cuts algorithm [13] was intended for segmenting an image into two regions. The grid graph representing pixels is transformed into a flow network by introducing a source and sink node. Each pixel node has an incoming edge from the source and an outgoing edge to the sink. Seed pixels are specified as belonging to either the background or foreground. Foreground seeds are severed from the sink node, and background seeds are severed from the source. By solving the max-flow/min-cut problem, a set of edges is found that partitions the graph into two. All pixels connected to the source are labelled as foreground, and vice versa.

#### 2.2.2. Random Walker

The random walker (RW) algorithm [14] was designed for multi-label image segmentation, rather than only foreground/background. Similar to graph cuts, a grid graph is used to represent the image. The edges between pixels are weighted from zero to one, representing the probability that a random walker on the graph will cross that edge. The weight of an edge u↔v is inversely proportional to the difference between the values of pixels *u* and *v*, i.e., a random walker is more likely to cross an edge between similar pixels. Each seed pixel is given a label from one to *k*.

The algorithm makes use of an equivalence theorem between random walks on a graph and electric potentials in a grid circuit. Each edge in the graph is represented by a resistance that is the inverse of the edge weight. For each label *i*, the seed pixels labelled *i* are given a unit potential while the other seeds are set to zero. The resulting potential at each unlabelled pixel *u* is the probability that a random walker beginning on *u* will first reach a seed pixel with label *i*. Once all *k* probabilities have been calculated, the pixel is assigned the label with the highest probability.

By representing the problem as an electric circuit, the image is segmented by solving a series of linear equations, rather than actually simulating a random walk.

#### 2.2.3. GrowCut

Like the RW algorithm, GrowCut [15] is designed to handle multi-label segmentation. However, it does not use an explicit graph structure to segment the image. Instead, the image is treated as a cellular automaton, which evolves over time. A cellular automaton has three basic properties: a non-empty state set *S*, a local neighbourhood *N* that defines adjacent pixels and a transition function $S^{N}\to S$ that determines the state set in the subsequent iteration. Seed pixels are labelled from one to *k* in the image. At each time step, a transition function is applied to pixels in the local neighbourhood of the seeds, which allows non-seed pixels to be labelled. The authors make the analogy of *k* competing bacteria cultures, which grow from their seed positions and attempt to claim new pixels as territory. The transition function is designed so that the competing populations eventually reach a stable configuration, segmenting the full image.

Our review of related works has shown the need for assisted segmentation of human depth datasets, and a means of achieving this via interactive segmentation. GrowCut and RW were both initially considered for this application. RW was eventually chosen for its speed of computation and tendency to produce smooth boundaries between regions. As described in the following section, it is used twice: once for segmenting the arms to create the layered graph and finally to produce the output segmentation.

## 3. Methodology

### 3.1. Grid Graph

The depth image is converted into an undirected grid graph, with one node for each pixel in the foreground. The pixel at row *i*, column *j* is connected to its four-neighbourhood in the image: the pixels at (i,j+1), (i,j−1), (i+1,j) and (i−1,j). The connection is represented by an edge in the grid graph. For an undirected edge u↔v, the weight is:

(1) $$w_{uv}=\exp\left(-\beta {\left(i_{u}-i_{v}\right)}^{2}\right)$$

where $i_{u}$ denotes the value at pixel *u*. This is the standard weighting function for RW [14]. In this case, $i_{u}$ is the depth at pixel *u*. The parameter β determines the significance of the difference in depth values. The ${\left(i_{u}-i_{v}\right)}^{2}$ values are first normalized across the image, as suggested in [14].

### 3.2. Arm Probability Matrix

We first aim to segment the two arm regions, as we assume these are the regions that are potentially occluding other parts of the body.

**Figure 1:**
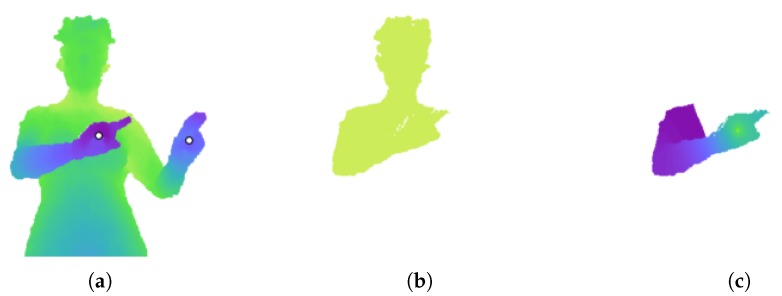
Effect of adding a dummy node. (**a**) Depth image. The white dots indicate the annotated hand pixels. (**b**) Probability values for the right hand, using the two hand pixels as seeds for the RW algorithm. The values are close to either zero or one. (**c**) Probability values after adding a dummy node to the graph. There is now a greater variance in the values.

As described in Section 2.2.2, the RW algorithm produces a probability matrix for each seed label. The algorithm is first run to obtain a probability matrix for each arm. The two hand pixels are used as seeds. Running RW using these two seed pixels, labelled 1 and 2, results in two probability matrices. However, if an arm is in front of the torso, the probability values can be skewed to be close to either zero or one. This can be seen in Figure 1. The depth difference of the right arm acts as a barrier to a random walker. As a result, even pixels on the head are given a probability of nearly one for belonging to the right hand.

**Figure 2:**
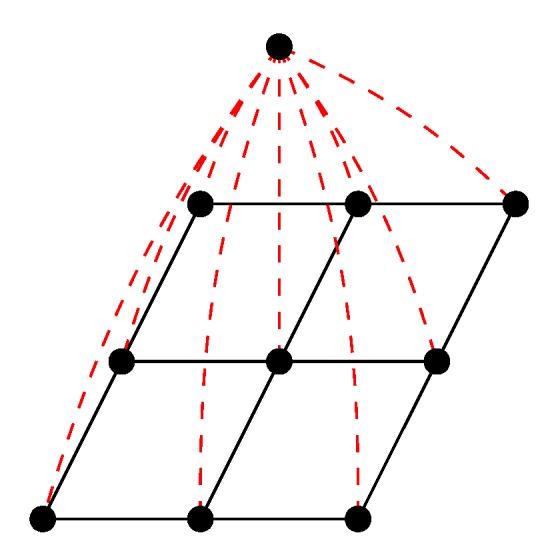
Diagram of a dummy node being added to the grid graph. Each node in the grid represents a pixel in the depth image. The dummy node is connected to each grid node by a single edge (dashed line).

To increase the variance in probability values, an extra ‘dummy’ node is added to the graph. This node is connected to every node in the grid graph by an edge with a small weight *w*, as shown in Figure 2. A value of $w=1\times 10^{-3}$ was determined through experiment. This represents the probability that a random walker will cross the edge and land on the dummy node. The RW algorithm is run on this new graph structure using three seed nodes: the two hand nodes and the dummy node, labelled 1 to 3. The output probability values for the hand nodes now have more variance, as evident in Figure 1. The new matrix *P* for hand *X* is used to segment arm *X* (left or right).

### 3.3. Arm Segmentation

If the arm is occluding the body, there will be a significant difference in depth between pixels on the arm and neighbouring pixels on the body. This will also be evident in the probability matrix *P* found in Section 3.2.

The probability values of *P* are clustered using the mean shift algorithm [16] with a Gaussian kernel. For efficiency, only a small sample of probability values is clustered. After sorting the probabilities, *n* uniformly-spaced values are taken from the sorted list and clustered with mean shift, resulting in *k* clusters. The value of *n* can be tuned by the user for their specific dataset. Mean shift automatically determines the number of clusters.

**Figure 3:**
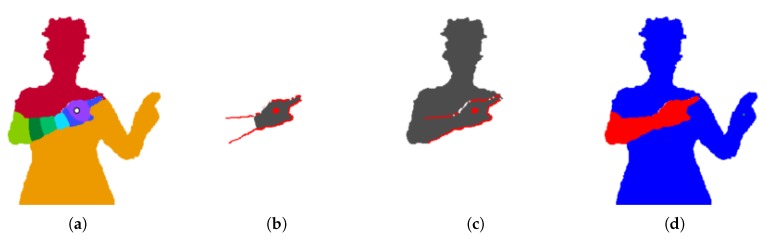
Segmenting the arms. (**a**) Segmented image resulting from clustering probability values of the right arm with mean shift. (**b**) Potential arm segment (grey) and strong gradient pixels (red). While this binary arm segment $B_{i}$ does not cut across the gradient, its complement ~*B_i_* does. This invokes a high cost. (**c**) Another potential arm segment. The segment *B_i_* cuts across the gradient, also invoking a high cost. (**d**) Final arm segments (the left segment is minuscule). Only the right arm is valid.

A new image $I_{seg}$ is the result of segmenting the probability matrix with mean shift. Each pixel in the foreground is assigned to the cluster with the closest centroid value. Figure 3 shows an example of this segmented image.

An iterative process is used to find the arm segment that minimizes a cost function, as explained below. Beginning with the segment on $I_{seg}$ corresponding to the highest cluster (i.e., the segment containing the hand pixel), segments are added in order of descending probability. At each iteration *i*, the full arm segment is the union of segments in $I_{seg}$ from one to *i*.

The image gradient captures information about the local change in pixel values [17]. It returns an (x,y) vector at each pixel location in an image. $I_{grad}$ is the magnitude of the image gradient of the probability matrix *P* (with all background pixels of *P* set to null). An example is shown in Figure 3. The highest values in $I_{grad}$ occur at sharp differences in pixel probability, corresponding to the depth difference caused by the arm occluding the body. The cost function for the arm segment uses this image gradient.

The binary arm segment $B_{i}$ is eroded with the structuring element for the four-neighbourhood.

(2) $$\mathrm{erosion}\left(B_{i}\right)=B_{i}⊖\left[\begin{array}{ccc} 0 & 1 & 0\\ 1 & 1 & 1\\ 0 & 1 & 0 \end{array}\right]$$

A new binary image $B_{union,\;i}$ is the union of the eroded $B_{i}$ and the erosion of its complement image, i.e., all image pixels not in $B_{i}$, including background pixels.

(3) $$B_{union,\;i}\;=\;\mathrm{erosion}\left(B_{i}\right)\;\cup \;\mathrm{erosion}(\sim B_{i})$$

The cost of $B_{i}$ is the mean of gradient values inside $B_{union,\;i}$.

(4) $$\cos t\left(B_{i}\right)=\mathrm{mean}\left(I_{grad}\left(B_{union,\;i}\right)\right)$$

In essence, an arm segment $B_{i}$ that is too small will cause ~*B_i_* to cut across a strong gradient line, while a *B_i_* that is too large will cut across a strong gradient line itself. This is evident in Figure 3. The segments are eroded to avoid the gradient values along their perimeters.

Algorithm 1 shows the pseudocode for the iterative process that segments the arm. It selects the arm segment that minimizes the cost defined in Equation (4). After the two arm segments have been found, there may be pixels that belong to both segments. Each of these pixels are assigned to the side with the higher RW probability.

**Algorithm 1** Arm segmentation.
1:Sort values of probability matrix *P*2:Select *n* evenly-spaced samples from sorted list3:$I_{seg}\leftarrow$ Image segmented by applying mean shift to probability samples4:$I_{grad}\leftarrow$ Image gradient of *P*


5:**function** ArmCost(*B*, $I_{grad}$)6:    $E_{1}\leftarrow$ erosion of binary image *B*7:    $E_{2}\leftarrow$ erosion of complement image ~*B*8:    $E_{union}\leftarrow E_{1}\cup E_{2}$9:    **return** $\mathrm{mean}\;(I_{grad}\left(E_{union}\right))$10:
**end function**



11:**procedure** SegmentArm($I_{seg}$, $I_{grad}$)12:    $C_{min}\leftarrow$ infinity13:    **for** *i* in *k* clusters **do**14:        $B_{i}\leftarrow$$⋃I_{seg}==\left\{1,\ldots ,i\right\}$15:        C←ArmCost($B_{i}$, $I_{grad}$)16:        **if** $C<C_{min}$ **then**17:           $C_{min}\leftarrow C$18:           $B_{arm}\leftarrow B_{i}$19:        **end if**20:    **end for**21:    **return** $B_{arm}$22:
**end procedure**



### 3.4. Validation of Arm Segments

Once the arms have been segmented, they are evaluated for evidence that they are occluding the body. An occluding arm segment is referred to as valid. In the case that an arm is not occluding the body, there can be an insignificant depth difference between the body and the arm.

$B_{arm}$ is the arm segment determined from Section 3.3. $F_{other}$ is the rest of the foreground *F*, including the other arm.

(5) $$F_{other}\;=\;F\;\cap \;\sim B_{arm}$$

$B_{inner}$ is the set of pixels in $B_{arm}$ that are in the four-neighbourhood of $F_{other}$. This set is found by dilating $F_{other}$ and taking the intersection with $B_{arm}$.

(6) $$B_{inner}=\left(F_{other}⊕\left[\begin{array}{ccc} 0 & 1 & 0\\ 1 & 1 & 1\\ 0 & 1 & 0 \end{array}\right]\right)\cap B_{arm}$$

**Figure 4:**
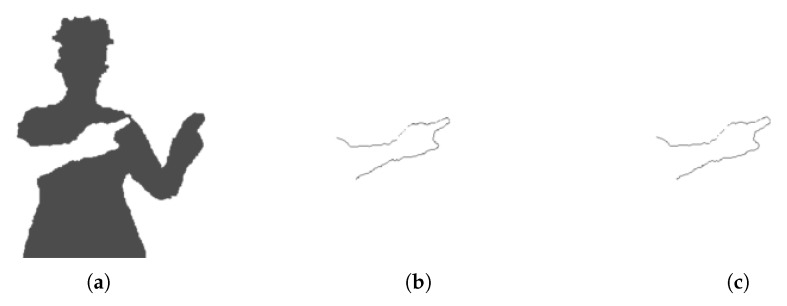
Relevant binary images. (**a**) $F_{other}$ is the set of foreground pixels outside the arm segment (in this case, the right arm). (**b**) $B_{inner}$ is the set of pixels inside the arm and neighbouring $F_{other}$. (**c**) $B_{outer}$ is the set of pixels inside $F_{other}$ and neighbouring the arm.

Similarly, $B_{outer}$ is the set of pixels in $F_{other}$ that are in the four-neighbourhood of $B_{arm}$. Figure 4 shows the inner and outer pixels for the right arm segment.

If the arm is occluding the body, there should be a significant difference between the depths of pixels in $B_{inner}$ and $B_{outer}$, and the outer depths should be greater than the inner. There should also be a significant probability difference. Let $D_{inner}$ be the set of depths of pixels in $B_{inner}$ and $P_{inner}$ be the set of probabilities. An arm segment is valid only if it is whole (i.e., only one connected component) and if it meets both of the following two conditions:

(7) $$\mathrm{median}\left(D_{outer}\right)-\mathrm{median}\left(D_{inner}\right)\geq 1$$

(8) $$\mathrm{median}\left(P_{inner}\right)-\mathrm{median}\left(P_{outer}\right)\geq 1\%$$

In the case shown in Figure 3, the right arm is valid and the left is not.

### 3.5. Layered Grid Graph

After the arms have been segmented, a new grid graph *G* is created with one to three ‘layers’: a base layer $G_{B}$, a right arm layer $G_{R}$ and a left arm layer $G_{L}$. Each layer is a distinct set of nodes. A layer is created for an arm only if the segment was found to be valid.

The purpose of this layered graph is to emulate a body with the arms outstretched, rather than occluding the mid-region. The base layer $G_{B}$ is a grid graph with one node for every foreground pixel, including the pixels that are in the right or left arm segments. A pixel inside binary arm segment $B_{arm,\;X}$ is represented by a node in $G_{B}$ and a node in $G_{X}$.

When all three layers are constructed, the graph *G* has $n_{G}$ nodes.

(9) $$n_{G}=n_{F}+n_{R}+n_{L}$$

where $n_{F}$ is the number of foreground pixels, $n_{R}$ is the number of pixels in arm segment $B_{arm,R}$ and $n_{L}$ is the number of pixels in arm segment $B_{arm,L}$.

In the regions of the arms, nodes in $G_{B}$ are given interpolated depth values, which are used to compute edge weights. This allows a segmentation algorithm to be unaffected by the sharp depth difference caused by occluding arms. To interpolate the depth values, every background (i.e., non-human) pixel is given the same depth, which is the median of all foreground depths. Then, the arm regions of the base layer are filled with inward interpolation. The edges of *G* are weighted using Equation (1).

### 3.6. Connecting Graph Layers

When the graph layers $G_{B}$, $G_{R}$ and $G_{L}$ are first constructed, there are no edges between layers. A set of nodes is selected that will connect *G* to $G_{R}$ and another set to connect *G* to $G_{L}$.

$I_{grad,\;inner}$ is the set of gradient values on $B_{inner}$ (from Equation 6).

(10) $$I_{grad,\;inner}=I_{grad}\left(B_{inner}\right)$$

**Figure 5:**
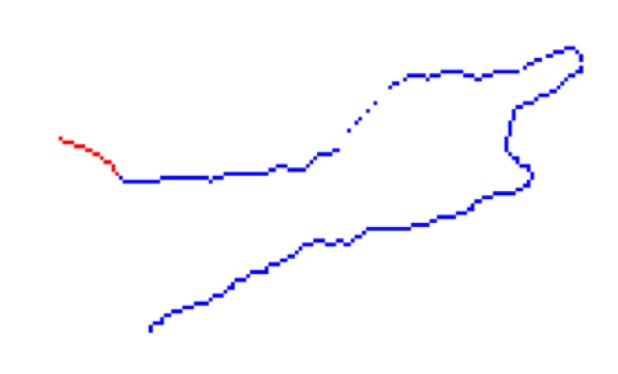
Pixels on the perimeter of an arm segment and adjacent to the rest of the foreground (binary image $B_{inner}$, also shown in Figure 4). The pixels used to connect graph layers (binary image $B_{connect}$) are shown in red.

Similar to the clustering in Section 3.3, the values of $I_{grad,\;inner}$ are clustered with mean shift. Along the inner pixels of the arm segment, the gradient is lowest where the arm connects to the body. $B_{connect}$ is the set of pixels in $B_{inner}$ corresponding to the lowest value cluster found by mean shift. These connecting pixels are shown in Figure 5.

Each pixel in $B_{connect,X}$ has two nodes associated with it: node *u* in the base graph $G_{B}$ and node *v* in the arm graph $G_{X}$. An edge u↔v is inserted with unit weight. This is repeated for each pixel $p\in B_{connect,X}$.

### 3.7. Assigning Body Parts to Layers

Every annotated part position must be assigned to its correct layer in the graph *G*. Each part is initially assumed to belong to the base layer of *G*. The forearm parts of arm *X* (e.g., hand and elbow) are assumed to be the only parts that can occlude the body; therefore, they are the only candidates for belonging to layer *X*. If the segmentation for arm *X* is valid, the hand of this arm must belong to layer *X* in the graph. The elbow is assigned to layer *X* only if its position is inside the arm segment $B_{arm,\;X}$.

### 3.8. Seed Nodes and Labels

In order to run an interactive segmentation algorithm on the graph, a subset of nodes must be specified as seeds. The provided body part positions are used as seed nodes, with labels one to $n_{parts}$.

More seed nodes can be added by drawing lines between adjacent body parts. Bresenham’s algorithm [18] is used to find the pixels that constitute a line between two image positions *A* and *B*. This line $L_{AB}$ is now split into $L_{A}$ and $L_{B}$, i.e., pixels in $L_{A}$ and $L_{B}$ are given labels *A* and *B*, respectively. The user specifies a value *r*, which is the ratio of the length of $L_{A}$ to the total length of $L_{AB}$. This allows the user to alter the size of the final part segment.

Each pixel in $L_{AB}$ is assigned to a node in the layered graph. The layers for each body part position have been found by the process described in Section 3.7. Each seed pixel with label *i* is assigned to the layer of part *i*. Thus, seed pixels on the image are converted to seed nodes in the layered graph.

### 3.9. Final Segmentation

Once the seed nodes and labels have been determined, an interactive image segmentation algorithm can be run on the layered graph *G*. The RW algorithm is used for the final segmentation. Every node in the graph is given a label by the algorithm. The final output image has the same size as the original depth image, and each pixel in the foreground receives a label. Pixels in arm segment *X* are represented by a node in $G_{B}$ and a node in $G_{X}$. The final label of these pixels is the label of the node in $G_{X}$. The label of the other node in $G_{B}$ is ignored.

## 4. Experimental Results

### 4.1. Datasets

Our method is tested on two public datasets of human depth images with segmented parts: “Human Limbs from RGBD data” [4] and “Human Depth Images with Body Part Labels” [5]. These are referred to as Datasets 1 and 2. In both datasets, only the foreground depth values are available.

Dataset 1 includes two actors in three video sessions, performing gestures in front of a Kinect v1 camera. Only the upper body is shown. The accompanying ground truth images have seven labels: torso, right/left upper arm, right/left lower arm and right/left hand. Each frame is a 640×480, 12-bit depth image. The three sessions have 236, 156 and 100 frames, respectively, for a total of 492 frames. Corresponding RGB images are included in the dataset, but our method is tested only on the depth images.

Our method is tested on one case from Dataset 2: sitting poses viewed from the front. This set contains nearly 5000 depth images of the full human body. Each frame is a 212×256 image, and 11 body parts are segmented.

### 4.2. Experiment Setup

The RW algorithm is performed using an open source MATLAB program written by the author of the algorithm [19]. In this implementation, the default value for the weighting parameter of Equation (1) is β=90. Our method uses this value of β for the arm segmentation and final segmentation.

Each input body part position is annotated using the ground truth labels of the test dataset. For each part *i*, the annotated part position is the foreground pixel closest to the centroid of all pixels labelled *i* (the pixel is not required to have truth label *i* itself).

To show that our method offers an improvement over generic interactive image segmentation, the standard RW algorithm is run on all frames with the annotated part positions as seed pixels, using the same β value.

#### 4.2.1. Dataset 1

Some additional pre-processing is used for this dataset. As an exception to the annotation rule defined above, the torso pixel is the mean position of the left and right upper arm pixels, whenever both of these arm parts exist. Some of the depth images include abnormally high depth values along the outline of the human figure, interfering with the RW algorithm. Therefore, the depth images are pre-processed to remove pixels with values greater than 2.5 metres. The same pixels are removed from the truth images.

**Figure 6:**
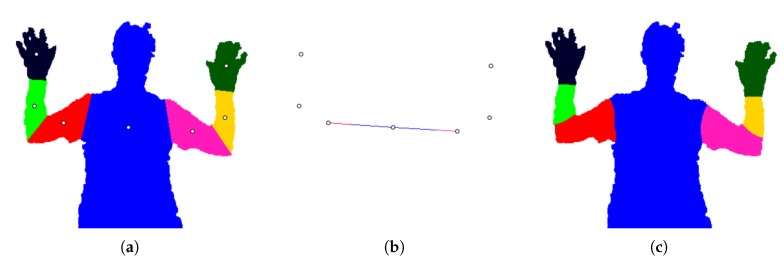
Segmenting with line seed pixels. (**a**) Ground truth image and part positions. (**b**) A line of seed pixels is drawn from the torso to each upper arm position. The first 2/3 of the line is labelled torso, and the remaining pixels are labelled L/R upper arm. Each other part *i* receives a single seed pixel. (**c**) Final segmentation.

Two versions of our method are tested. The first (referred to as ‘our method, no line’) uses only the seven part positions as seeds for the segmentation. The second (referred to as ‘our method, with line’) adds a line of seed pixels between the torso and the two upper arm positions, as per Section 3.8. The ratio *r* is set to 2/3 for all frames. This is intended to limit the size of the upper arm segments. An example is shown in Figure 6.

The results of our method are also compared to the best performance of two algorithms tested on the same dataset [4,10].

#### 4.2.2. Dataset 2

Because of the relative positions of the ground truth labels for this dataset, it was decided that adding a line of seed pixels was unnecessary. Thus, only the simple version of our method is tested on this dataset, using the 11 annotated part positions as seed pixels.

### 4.3. Evaluation

Two metrics are used to evaluate our method: the per-class accuracy PC and the Jaccard index JI. These are calculated as described in [20]. The confusion matrix C is computed using the ground truth label image and the predicted label image. $C_{ij}$ is the number of pixels with truth label *i* and predicted label *j*. The matrix has dimensions L×L, where *L* is the number of labels.

The per-class accuracy for label *i* is the number of pixels correctly labelled *i* over the total number of pixels labelled *i*. This metric has been previously used to report results on Dataset 1 [4,10].

(11) $${\mathrm{PC}}_{i}=\frac{C_{ii}}{\sum_{j}C_{ij}}$$

The Jaccard index for label *i* is the number of pixels labelled *i* in both images over the number of pixels labelled *i* in either image, i.e., the intersection over union. This has been the standard evaluation metric for the PASCAL challenge since 2008 [20], which is a benchmark competition for object classification and detection [21].

(12) $${\mathrm{JI}}_{i}=\frac{C_{ii}}{-C_{ii}+\sum_{i}C_{ij}+\sum_{j}C_{ij}}$$

#### Pairwise Comparisons

To evaluate one method against another, the mean PC and JI values are calculated for each frame and by both methods. Let $V_{A}$ and $V_{B}$ be the mean metric values for a single frame segmented by methods *A* and *B*, respectively. A win for method *A* is recorded when $V_{A}>V_{B}$, a loss when $V_{A}<V_{B}$ and a tie when $V_{A}=V_{B}$. The differential is $V_{A}-V_{B}$.

### 4.4. Results

#### 4.4.1. Dataset 1

**Figure 7:**
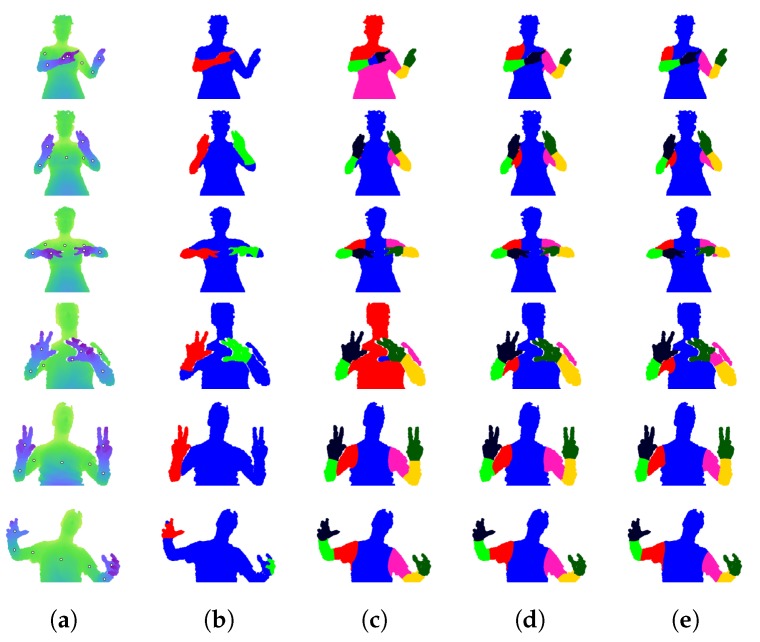
Dataset 1 [4]: Qualitative results. (**a**) Depth image with annotated part positions. (**b**) Arm segmentation. (**c**) Standard RW segmentation. (**d**) Our method without line seed pixels. (**e**) Our method with line seed pixels.

Qualitative results are shown in Figure 7. The first column shows the depth images and annotated part positions, and the second shows the arm segmentations. The following columns display the three segmentation methods that were tested on Dataset 1.

Table 1 shows the mean Jaccard index for each body part. We use the same abbreviations as [4]: L, left; R, right; U, upper; W, lower. Both versions of our method have higher overall averages (82.27% and 86.01%) than the standard RW algorithm (80.31%). Adding a line of seed pixels, as described in Section 4.2, results in a higher average, with the largest gains in the upper arm segments.

The mean per-class accuracy is shown in Table 2. In this case, adding line seed pixels causes a slight decrease in performance. In terms of the overall average, both versions of our method outperform the standard RW, as well as the two cited algorithms.

Table 3 and Table 4 show the results of pairwise comparisons. The total differential is the sum of differentials over all frames, i.e., a positive value indicates a net advantage of using method *A* over method *B*. For both metrics, the two versions of our method outperform the standard RW algorithm. Examining the total differentials, adding line seeds causes a slight decrease in per-class accuracy (−0.99%), but a large increase in the Jaccard index (18.40%).

#### 4.4.2. Dataset 2

**Figure 8:**
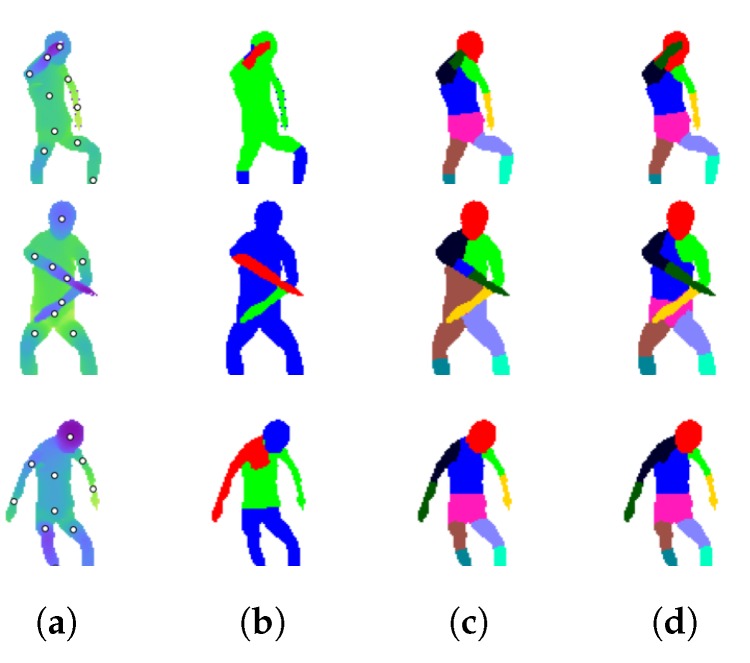
Dataset 2 [5]: Qualitative results. (**a**) Depth image with annotated part positions. (**b**) Arm segmentation. (**c**) Standard RW segmentation. (**d**) Our method.

Qualitative results for Dataset 2 are shown in Figure 8. Similar to Figure 7, the first column shows depth images, and the second shows arm segmentations. These are followed by the results of RW and our method.

Table 5 shows the Jaccard index for our method and the standard RW algorithm on Dataset 2. There is a slight increase in the overall average, from 77.31% to 78.45%. The per-class results in Table 6 also show a slight increase in the overall average, from 90.31% to 90.60%. The pairwise comparison in Table 7 shows that our method outperforms RW on a majority of frames (3002 wins). While the comparison shows more losses than wins for the per-class metric, the total differential is still positive, indicating that the wins were by a greater margin than the losses.

#### 4.4.3. Analysis

As seen in the first and fourth row of Figure 7, the RW algorithm greatly fails when the torso position is located on a hand pixel. This problem is averted by our method, because the torso seed node is located on the lower graph layer, underneath the arm. Most of the rows demonstrate that adding a line of seeds reduces the size of the upper arm segments.

The first two rows of Figure 8 are examples of our method outperforming RW, while the third is an example of similar results. The first row performs well because the right arm is made into a graph layer, separating it from the head. In the RW figure, the right forearm is incorrectly labelled as the head. In the third row, the seed pixel for the hip is cut off from the rest of the midsection by the left arm. Our method allows the segment to spread further because the left arm is made into a graph layer.

Connecting graph layers (Section 3.6) allows the final segmentation algorithm to propagate a label across different layers. The first row of Figure 7 shows a right arm segment that is differently shaped than the final right arm parts. By connecting the graph layers, the upper arm label (red) can spread into the arm segment, reducing the size of the lower arm part (green).

Between the two versions of our method tested on Dataset 1, there is a trade-off between the two evaluation metrics. We hypothesize that adding line seed pixels greatly increases the Jaccard index because it limits the size of the upper arm segments. The left and right upper arm values increase from 65.04% and 66.62% to 74.98% and 75.84%, respectively, when the line seeds are added (Table 1). Since the per-class accuracy measures the ratio of pixels correctly labelled *i* to the total number of pixels labelled *i*, an overly large arm segment will still have a high accuracy. If the torso pixels are misclassified as upper arm, the per-class accuracy does not greatly reduce because the torso is so large by comparison. For this reason, we consider the Jaccard index to be a more discriminative metric and conclude that adding a line of seed pixels is overall advantageous to our method.

The results indicate that our method offers superior performance to the standard RW algorithm. By creating a layered graph structure, there is an increase in both the Jaccard index and the per-class accuracy. Our method also outperforms two state of the art algorithms [4,10] on the same dataset. However, it should be noted that these other algorithms addressed the more difficult problem of segmentation without annotated part positions.

## 5. Conclusions

We have presented a graph-based approach to segmenting human parts from depth images, given the image positions of each part. We propose a layered graph structure that can handle self-occlusion by the arms. The provided part positions are used to determine seed nodes and labels for a standard interactive segmentation algorithm. This work is intended to facilitate the labelling of human segmentation datasets, which can be used for training and testing new algorithms.

Future work in this area could aim to optimize the two β values for segmentation. While our method only used the default value of β=90, two different β values can be used for segmenting the arms and final parts. Other interactive segmentation algorithms can be tested on the same layered graph structure, and the full method can be tested on other types of images, such as RGB or RGB-D. There is also room for improving the method of segmenting the arms and testing for signs of occlusion.

The main conclusion from this study is that a layered graph representation of an image can improve the performance of an interactive segmentation algorithm, when segmenting body parts with a small number of seed pixels. This has been demonstrated on two depth datasets of humans from a frontal perspective. To encourage further research on this topic, our implementation will be made publicly available at https://github.com/ajhynes7.

## Figures and Tables

**Table 1:** Dataset 1 [4]: mean Jaccard index in % over all frames. U, upper; W, lower.

	Torso	LU arm	LW arm	L hand	RU arm	RW arm	R hand	Average
RW	91.83	60.31	82.31	92.79	60.65	82.48	91.77	80.31
Our method, no line	95.81	65.04	81.85	92.69	66.62	81.63	92.25	82.27
Our method, with line	97.73	74.98	84.81	92.55	75.84	84.11	92.03	86.01

**Table 2:** Dataset 1 [4]: mean per-class accuracy in % over all frames.

	Torso	LU arm	LW arm	L Hand	RU arm	RW arm	R hand	Average
Hernández-Vela et al. [4]	98.44	78.93	84.38	88.32	82.57	88.85	93.86	87.91
Liang et al. [10]	94.10	93.57	90.43	87.31	93.13	87.84	85.79	90.31
RW	92.29	85.06	95.71	96.69	85.65	95.46	95.75	92.37
Our method, no line	96.13	87.46	95.66	96.02	88.59	95.45	95.53	93.55
Our method, with line	98.65	87.67	92.84	95.92	91.40	91.47	95.48	93.35

**Table 3:** Dataset 1 [4]: pairwise comparison of methods with the Jaccard index. A win indicates that method *A* outperformed method *B* on one frame.

Method A	Method B	Wins	Losses	Ties	Total Differential (%)
Our method, no line	RW	290	165	37	9.67
Our method, with line	RW	432	60	0	28.06
Our method, with line	Our method, no line	436	55	1	18.40

**Table 4:** Dataset 1 [4]: pairwise comparison of methods with per-class accuracy.

Method A	Method B	Wins	Losses	Ties	Total Differential (%)
Our method, no line	RW	249	207	36	5.85
Our method, with line	RW	229	263	0	4.86
Our method, with line	Our method, no line	215	276	1	−0.99

**Table 5:** Dataset 2 [5]: mean Jaccard index in % over all frames.

	Torso	Head	LU arm	RU arm	Hip	LW arm	RW arm	LU leg	RU leg	LW leg	RW leg	Avg
RW	60.19	89.58	69.50	65.21	56.82	88.71	89.95	79.36	75.56	88.68	86.87	77.31
Our method	64.74	90.98	71.80	67.32	57.06	89.81	90.54	79.28	75.09	89.15	87.20	78.45

**Table 6:** Dataset 2 [5]: mean per-class accuracy in % over all frames.

	Torso	Head	LU arm	RU arm	Hip	LW arm	RW arm	LU leg	RU leg	LW leg	RW leg	Avg
RW	60.71	99.42	97.85	96.86	94.35	89.69	90.99	94.79	92.37	89.13	87.20	90.31
Our method	65.59	98.23	97.77	95.79	94.71	90.85	91.82	93.73	91.10	89.53	87.47	90.60

**Table 7:** Dataset 2 [5]: pairwise comparison of methods.

Metric	Method A	Method B	Wins	Losses	Ties	Total Differential (%)
Jaccard	Our method	RW	3002	1839	83	53.49
Per-class	Our method	RW	2306	2528	90	12.63

## Abbreviations

The following abbreviations are used in this manuscript:

RW	Random walker
HOG	Histogram of oriented gradients

References

## References

[1] Charles J., Everingham M. Learning shape models for monocular human pose estimation from the Microsoft Xbox Kinect. Proceedings of the Conference on 2011 IEEE International Computer Vision Workshops (ICCV Workshops).

[2] Chandra S., Tsogkas S., Kokkinos I. Accurate Human-Limb Segmentation in RGB-D Images for Intelligent Mobility Assistance Robots. Proceedings of the 2015 IEEE International Conference on Computer Vision Workshop (ICCVW).

[3] Roccetti M., Marfia G., Semeraro A. (2012). Playing into the wild: A gesture-based interface for gaming in public spaces. J.Visual Commun. Image Represent..

[4] Hernández-Vela A., Zlateva N., Marinov A., Reyes M., Radeva P., Dimov D., Escalera S. Graph cuts optimization for multi-limb human segmentation in depth maps. Proceedings of the 2012 IEEE Conference on Computer Vision and Pattern Recognition (CVPR).

[5] Nishi K., Miura J. (2017). Generation of human depth images with body part labels for complex human pose recognition. Pattern Recognit..

[6] Sánchez D., Ortega J.C., Bautista M.Á., Escalera S. (2013). Human body segmentation with multi-limb error-correcting output codes detection and graph cuts optimization. Iberian Conference on Pattern Recognition and Image Analysis.

[7] Xia F., Wang P., Chen L.C., Yuille A.L. (2015). Zoom Better to See Clearer: Human Part Segmentation with Auto Zoom Net. CoRR.

[8] Hernández-Vela A., Reyes M., Ponce-López V., Escalera S. (2012). GrabCut-Based Human Segmentation in Video Sequences. Sensors.

[9] Madadi M., Escalera S., Gonzalez J., Roca F.X., Lumbreras F. (2015). Multi-part body segmentation based on depth maps for soft biometry analysis. Pattern Recognit. Lett..

[10] Liang H., Yuan J., Thalmann D. (2014). Parsing the hand in depth images. IEEE Trans. Multimed..

[11] Shotton J., Fitzgibbon A., Cook M., Sharp T., Finocchio M., Moore R., Kipman A., Blake A. Real-time human pose recognition in parts from single depth images. Proceedings of the Conference on Computer Vision and Pattern Recognition (CVPR).

[12] Hernández-Vela A., Primo C., Escalera S. Automatic user interaction correction via multi-label graph cuts. Proceedings of the 2011 IEEE International Conference on Computer Vision Workshops (ICCV Workshops).

[13] Boykov Y.Y., Jolly M.P. Interactive graph cuts for optimal boundary & region segmentation of objects in ND images. Proceedings of the Eighth IEEE International Conference on Computer Vision (ICCV 2001).

[14] Grady L. (2006). Random walks for image segmentation. IEEE Trans. Pattern Anal. Mach. Intell..

[15] Vezhnevets V., Konouchine V. (2005). GrowCut: Interactive multi-label ND image segmentation by cellular automata. Proc. Graph..

[16] Comaniciu D., Meer P. (2002). Mean shift: A robust approach toward feature space analysis. IEEE Trans. Pattern Anal. Mach. Intell..

[17] Gonzalez R.C., Woods R.E. (2002). Digital image processing.

[18] Bresenham J.E. (1965). Algorithm for computer control of a digital plotter. IBM Syst. J..

[19] Grady L. Software. http://leogrady.net/software/.

[20] Csurka G., Larlus D., Perronnin F. What is a good evaluation measure for semantic segmentation?. Proceedings of the British Machine Vision Conference (BMVC 2013).

[21] Everingham M., Van Gool L., Williams C.K., Winn J., Zisserman A. (2010). The pascal visual object classes (voc) challenge. Int. J. Comput. Vis..

